# Supplementary material for: Yiai Fuzheng decoction inhibits triple-negative breast cancer by remodeling the immune microenvironment
Source: Front Immunol. 2025 Sep 30;16:1615631. doi: 10.3389/fimmu.2025.1615631 (PMC12518410; doi:10.3389/fimmu.2025.1615631)
Supplement: Supplementary file 2 [file DataSheet1.zip › Supplementary File 2/Treatment/D_vs_H/07_Pathway_Analysis/Enrichment_Analysis/smpdb_pathway/Network.pdf]

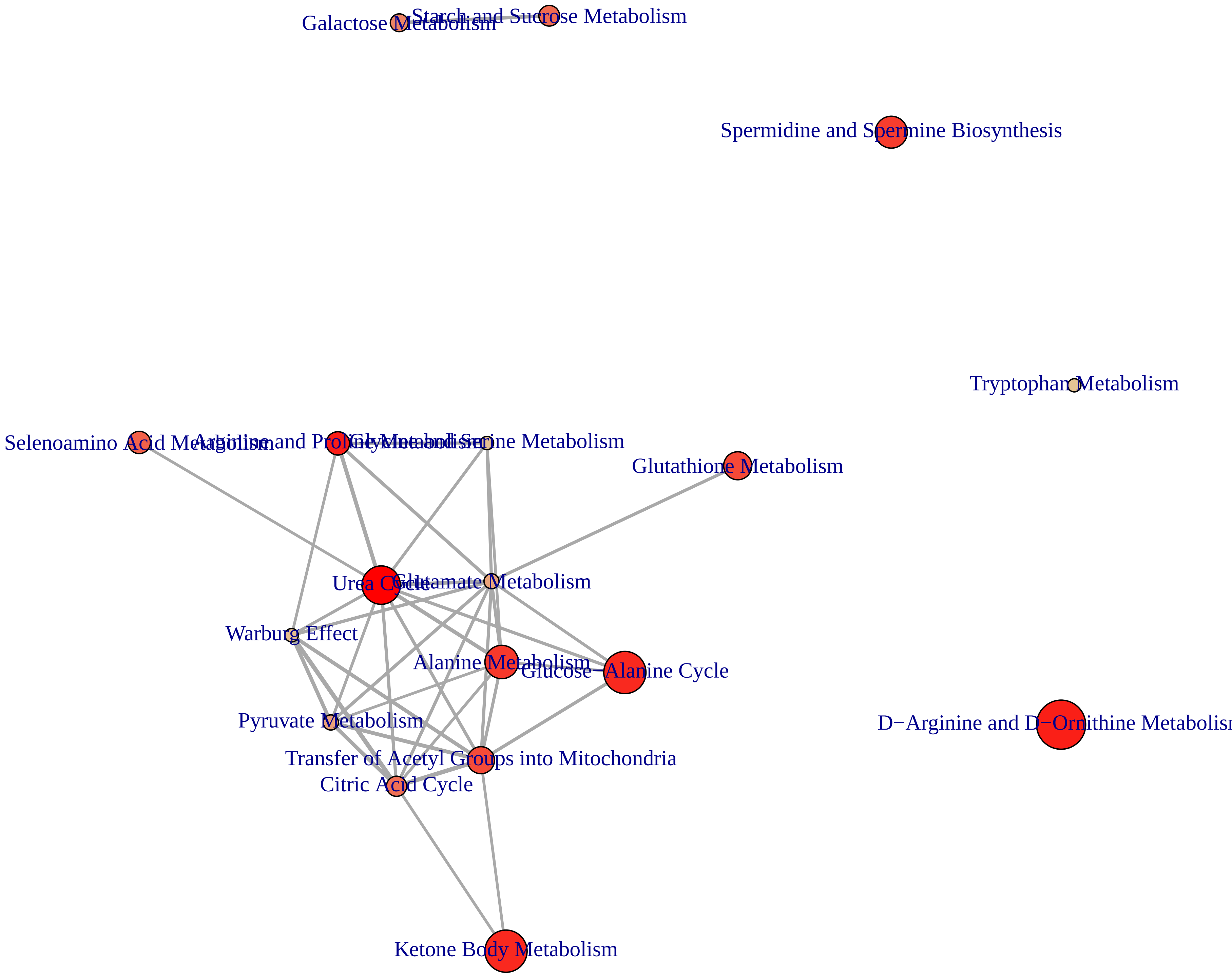
Starch and Sucrose Metabolism
Galactose Metabolism
Spermidine and Spermine Biosynthesis
Tryptophan Metabolism
Selenoamino Acid Metabolism
Arginine and Proline Metabolism
Glycine and Serine Metabolism
Glutathione Metabolism
Urea Cycle
Glutamate Metabolism
Warburg Effect
Alanine Metabolism
Glucose−Alanine Cycle
Pyruvate Metabolism
D−Arginine and D−Ornithine Metabolis
Transfer of Acetyl Groups into Mitochondria
Citric Acid Cycle
Ketone Body Metabolism
